# Sclerosing pneumocytoma originated in the middle mediastinum: a case report

**DOI:** 10.1186/s44215-023-00062-1

**Published:** 2023-08-10

**Authors:** Chihiro Yoshida, Mototsune Kakizaki, Chang Sung Soo, Taku Okamoto

**Affiliations:** 1 Department of General Thoracic Surgery, Kochi Health Sciences Center, 2125-1 Ike Kochi, 781-8555 Kochi City, Japan; 2 Department of Diagnosis of Pathology, Kochi Health Sciences Center, Kochi, Japan

**Keywords:** Sclerosing pneumocytoma, Mediastinal tumor, Video-assisted thoracic surgery

## Abstract

**Background:**

Sclerosing pneumocytoma is a benign tumor that occurs mostly in the lungs and very rarely in the middle mediastinum. It may be difficult to diagnose, as its radiological features and histologic heterogeneity mimic malignancy; therefore a histopathologic examination is required to establish a definitive diagnosis.

**Case presentation:**

The patient was a 52-year-old woman who was found to have a tumor in the middle mediastinum on a chest CT scan and was referred to our hospital for resection. We performed a resection of the mediastinal tumor, assuming it was thymoma or teratoma, via video-assisted thoracic surgery. Histopathological analysis of the mediastinal tumor revealed it to be a sclerosing pneumocytoma.

**Conclusions:**

Herein, we presented a case of a very rare sclerosing pneumocytoma originating from the middle mediastinum where video-assisted thoracic surgery allowed for minimally invasive resection and histopathological diagnosis of the tumor. Such procedures should be considered as the histopathological diagnosis of the tumor may help determine the optimal treatment options.

## Background

Sclerosing pneumocytoma (SP) is a rare benign tumor that most commonly occurs as a solitary tumor in the lung. It is extremely rare for it to originate isolated from the middle mediastinum. Previous studies have suggested that this neoplasm originates from the proliferation of type 2 alveolar lung cells and multipotent primitive respiratory epithelium. Additionally, it commonly occurs in middle-aged Asian women and has a benign course with good prognosis and no recurrence [1, 2]. This rare, benign disease can sometimes be difficult to diagnose because of its radiological features and histologic heterogeneity, which mimic malignant tumors [3]. Here, we present a case where video-assisted thoracic surgery (VATS) enabled the diagnosis and treatment of mediastinal SP.

## Case presentation

**Fig. 1:**
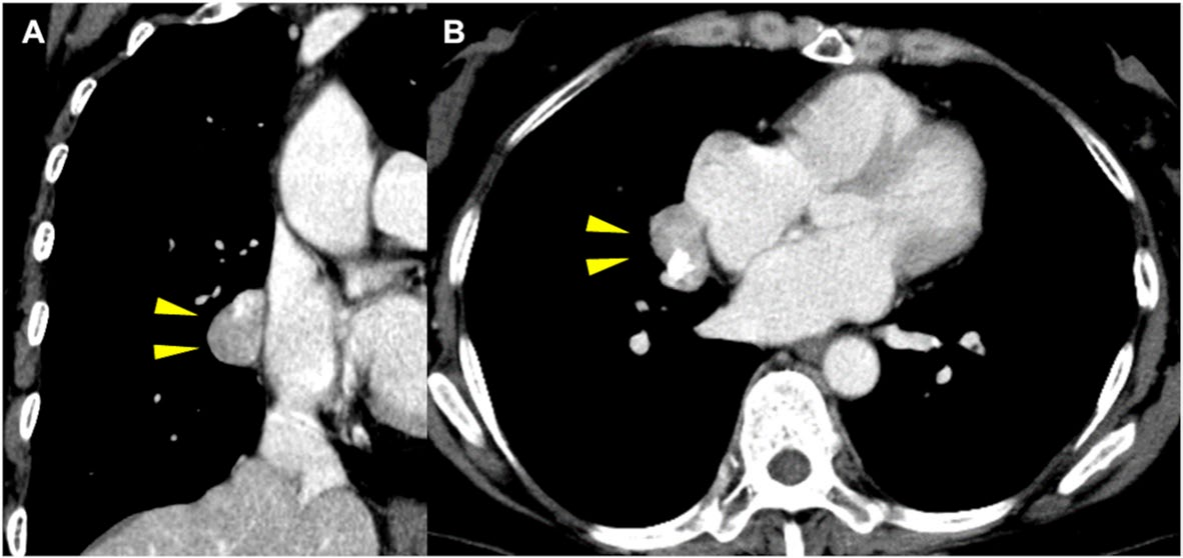
A contrast-enhanced CT image. **A**, **B** A contrast-enhanced CT image of the chest highlights a 3 cm heterogeneous mass with calcification in the mediastinum. Yellow triangles indicate the tumor

A 52-year-old female visited us for further investigation of a mass lesion observed in the area of the right atrium on a chest X-ray. The patient reported no significant medical history, and they had no symptoms of a respiratory disorder. Their physical examination upon admission was unremarkable and their laboratory results showed no abnormalities. Chest computed tomography (CT) showed a mass lesion, measuring 30 mm at the largest diameter, located in the middle mediastinum, which was homogeneous and showed strong enhancement with calcification (Fig. 1). CT showed that there were no other tumors in the lungs or surrounding tissues and no evident lymphadenopathy. The lesion showed a low signal on T1-weighted magnetic resonance imaging and a low and heterogeneous signal on T2-weighted fat-saturation imaging. Based on these findings, we suspected a thymoma or teratoma; therefore, we decided on surgical resection of the tumor to determine the diagnosis and treatment.

**Fig. 2:**
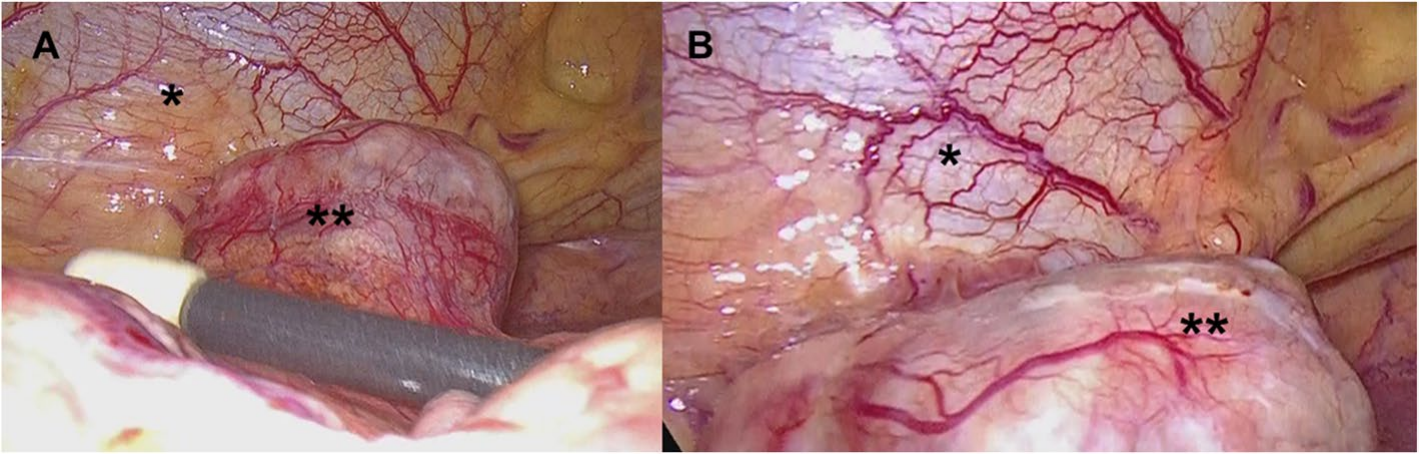
Intraoperative findings. **A** The tumor was located on the pericardium slightly posterior to the phrenic nerve. **B** The pedicle of the tumor was connected to the mediastinal pleura. Single asterisk (*) indicate heart with pericardium; double asterisks (**) indicate tumor

**Fig. 3:**
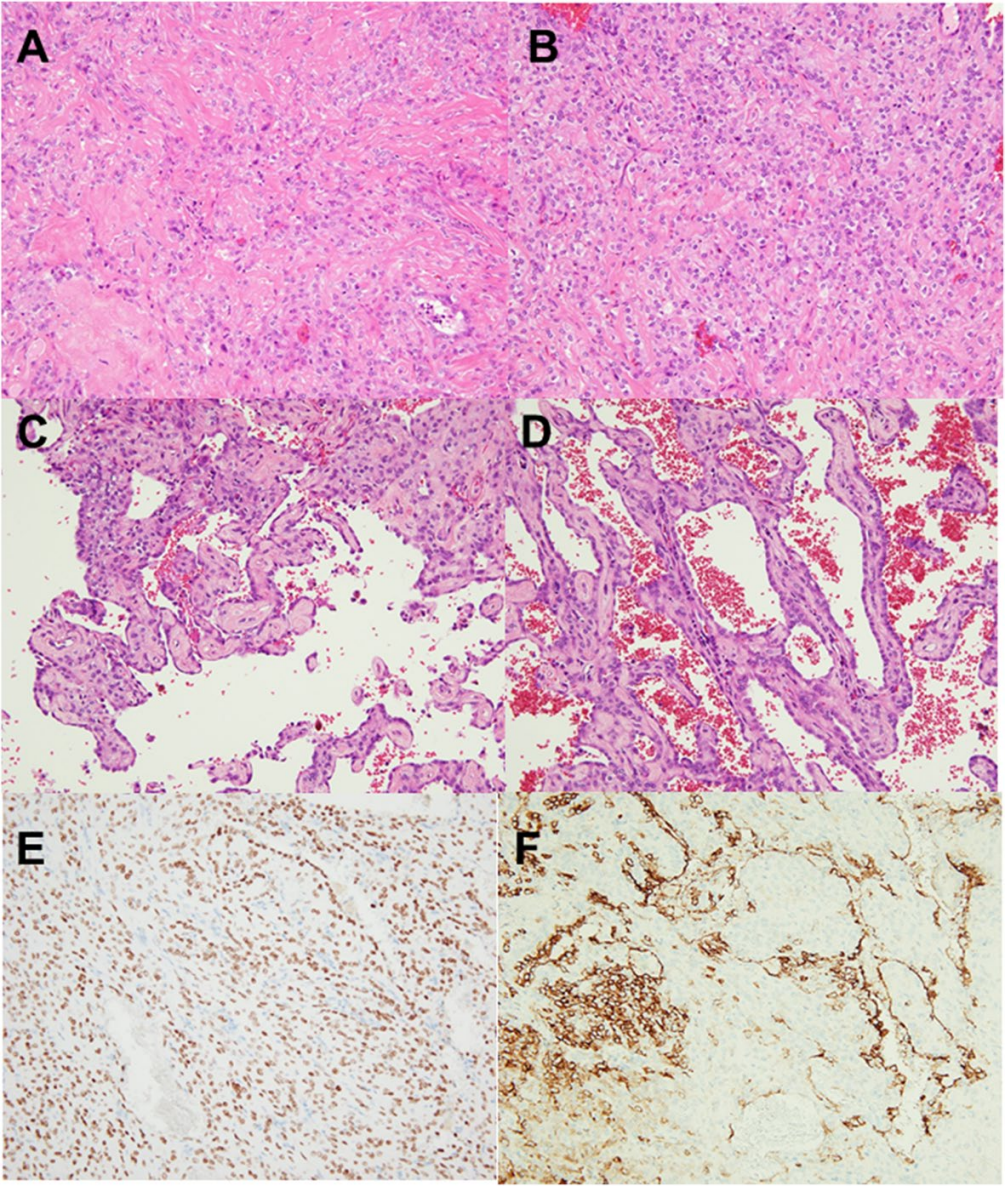
The histological and immunohistochemical images. **A** Histological features of sclerosing pneumocytoma; sclerotic areas (hematoxylin–eosin, 20 ×). **B** Solid areas (hematoxylin–eosin, 20 ×). **C** Papillary areas (hematoxylin–eosin, 20 ×). **D** Hemorrhagic areas (hematoxylin–eosin, 20 ×). **E** TTF-1 positive in cuboidal surface cells and stromal round cells (TTF-1 20 ×). **F** EMA immunolabeling positive in both cell types (EMA 20 ×)

Video-assisted thoracoscopic resection of the tumor was performed under general anesthesia. The patient was placed in the left semi-recumbent position, with the ipsilateral arm lifted above the chest, for port placement in triple-port surgery. Two ports were made in the fourth and sixth intercostal spaces with 5-mm trocars. One port was made in the third intercostal space with a 10-mm trocar and a thoracoscope was inserted. The tumor was located on the pericardium, slightly posterior to the phrenic nerve (Fig. 2) and had pedicles in the mediastinal pleura suggesting its origin. The tumor was carefully excised from the mediastinal pleura. The operation time was 90 min, and blood loss was 10 ml. A rapid intraoperative diagnosis did not reveal thymoma or teratoma; frozen section analysis showed no malignant findings. A further diagnosis could not be determined Fig. 3.

On microscopic examination, the lesions showed a combination of cuboidal cells lining the papillary structure and interstitial round cells composing the core of the papillary and solid areas. There were cuboidal surface cells found lining ectatic hemorrhagic spaces as hemorrhagic pattern. In addition, mast cells were conspicuous. Immunohistochemically, cuboidal cells were positive for thyroid transcription factor (TTF-1), epithelial membrane antigen (EMA), PAN-CK (AE1/AE3), napsin A, and surfactant protein A. Stromal cells were also positive for TTF-1 and EMA but negative for napsin A and surfactant protein A. Estrogen receptor and progesterone receptor positivity could be observed in some cells. Although solitary fibrous tumor and mesenchymal tumor were also differentiated from histological morphology and site of occurrence, both cuboidal cells and stromal cells were negative for Cytokeratin (CK5/6), CD34, Calretinin, WT-1, D2-40, and Desmin. Ki67 proliferation index was very low. Based on these results, sclerosing pulmonary cell tumor was diagnosed. The postoperative course was uneventful. The patient received no additional treatment and has been followed for six months without recurrence.

## Discussion

SP is a rare benign pulmonary tumor most often occurring in middle-aged and older women [3]. The prognosis after complete resection is good, even though rare cases of regional lymph node metastasis have been reported. In the literature, several unusual presentations of SP have been reported, such as multifocal lesions (4%), as well as endobronchial (1%), pleural (4%), and mediastinal (1%) localizations. On CT images, it appears as a firm, well-defined circular or oval mass with low CT values. Additionally, it has been shown that 30% of cases have calcifications within the sclerotic area of SP [4]. Because this case presented as a mediastinal mass, we did not initially consider the diagnosis of SP. However, careful review of the surgical findings revealed that this lesion originated in the periphery of the lung and protruded from the visceral pleural surface, giving the initial impression of an extrapulmonary origin.

It can be challenging to accurately diagnose SP, and it is often misdiagnosed as lung adenocarcinoma, especially on fine-needle aspiration and intraoperative frozen sections [5–7]. Cuboidal surface cells and interstitial round cells are key features of SP [8]. SP has characteristic histopathological features, usually displaying a mixed solid, papillary, hemorrhagic, and/or sclerotic pattern and contain two distinct cellular components: round cells and surface cells. Previous studies using laser microdissection and clonal analysis have demonstrated that round and surface cells exhibit a uniform pattern of monoclonality, suggesting that both cells may originate from a common progenitor [8, 9]. The four distinct growth patterns and dual cell types that may appear in different proportions are key pathological features of SP. In addition to the four growth patterns, foam cells foci, mixed cell components and hypercellular papillary core provide diagnostic clues for SP [4, 10, 11]. However, it is difficult to diagnose SP when a mediastinal tumor has been taken into consideration in the differential diagnosis, as in this case.

VATS can be safely used for mediastinal dissection [12, 13], and its role as a diagnostic modality for mediastinal masses has already been established. In this rare case of mediastinal SP, we were able to accurately diagnose and properly treat the patient by performing a minimally invasive resection via VATS. Before surgery, we had confirmed by CT that there were no lymph node metastases. If the tumor was resected by VATS and the intraoperative histology revealed that the tumor was a malignancy, such as thymic cancer, the strategy was to dissect and sample the lymph nodes around the tumor and the anterior mediastinal lymph nodes. Ultimately, lymph node dissection was not performed because the intraoperative histological examination indicated no malignancy. VATS appears to be a useful method for diagnosis and determining the appropriate treatment strategy in mediastinal tumors.

In conclusion, surgery was performed for a very rare SP of mediastinal origin. Surgical resection of an isolated mediastinal tumor may allow a histologic diagnosis and help determine appropriate treatment options.

## Data Availability

We confirm that the data supporting the findings of this study are available within the article and its supplementary materials.

## Abbreviations

CT: Computed tomography
EMA: Epithelial membrane antigen
SP: Sclerosing pneumocytoma
TTF-1: Thyroid transcription factor
VATS: Video-assisted thoracic surgery
